# Educational outcomes in childhood cancer survivors: A Scotland-wide record-linkage study of 766,217 schoolchildren

**DOI:** 10.1371/journal.pone.0286840

**Published:** 2023-07-26

**Authors:** Nicholas Baughan, Jill P. Pell, Daniel F. Mackay, David Clark, Albert King, Michael Fleming

**Affiliations:** 1 School of Medicine, Dentistry and Nursing, University of Glasgow, Glasgow, United Kingdom; 2 School of Health and Wellbeing, University of Glasgow, Glasgow, United Kingdom; 3 Public Health Scotland, Edinburgh, United Kingdom; 4 ScotXed, Scottish Government, Edinburgh, United Kingdom; University of Oxford, UNITED KINGDOM

## Abstract

**Background:**

A cancer diagnosis during childhood greatly disrupts the lives of those affected, causing physical and psychological challenges. We aim to investigate educational outcomes among schoolchildren with a previous cancer diagnosis compared to their peers.

**Methods:**

Individual records from four national education databases and three national health databases were linked to construct a cohort of all singleton schoolchildren born in Scotland attending Scottish local-authority schools between 2009–2013. Pupils previously diagnosed with any cancer, haematological cancers, and central nervous system (CNS) cancers, were compared to their unaffected peers with respect to five educational outcomes: special educational need (SEN), absenteeism, school exclusion, academic attainment, and unemployment. Analyses were adjusted for sociodemographic and maternity factors and chronic conditions.

**Results:**

Of 766,217 pupils, 1,313 (0.17%) had a previous cancer diagnosis. Children with any cancer had increased odds of SEN (OR 3.26, 95% CI 2.86–3.71), absenteeism (IRR 1.82, 95% CI 1.70–1.94), and low attainment (OR 2.15, 95% CI 1.52–3.03) compared to their peers. Similar findings were observed for haematological (SEN OR 2.62, 95% CI 2.12–3.24; absenteeism IRR 2.04, 95% CI 1.85–2.25; low attainment OR 2.17, 95% CI 1.31–3.61) and CNS (SEN OR 6.44, 95% CI 4.91–8.46; absenteeism IRR 1.75, 95% CI 1.51–2.04; low attainment OR 3.33, 95% CI 1.52–7.30) cancers. Lower exclusions were observed among children with any cancer (IRR 0.51, 95% CI 0.31–0.83) and CNS cancer (IRR 0.20, 95% CI 0.06–0.61). No associations were observed with unemployment.

**Conclusions:**

This study highlights the wider impacts of childhood cancer on educational outcomes. These children need to be supported, as poor educational outcomes can further impact later health.

## Background

Around 130 children under the age of 15 years are diagnosed with cancer each year in Scotland, most of whom have haematological cancers (e.g., leukaemia, lymphoma) or cancers of the central nervous system (CNS) [1]. A cancer diagnosis during childhood greatly disrupts the lives of those affected, causing physical and psychological challenges not experienced by peers. Whilst formal cancer treatments can last several years [2], longer term adversities include the threat of cancer relapses, and the impact of the disease and treatment on malnutrition, growth, mental health, and even fertility [3–5].

In Scotland, overall five-year cancer survival among children diagnosed between 2009–2013 was 83% (95% CI 80.4%–86.4%), therefore most will experience long-term consequences of cancer after leaving school [1]. Achieving a good education is a key wider determinant of health, influencing employment, wealth, standard of living, and overall wellbeing [6]. Therefore, identifying factors impacting educational outcomes is important to guide policy development, improve population health, and reduce health inequities. Whilst children with cancer face poorer health outcomes as a direct consequence of the condition, reduced educational performance may indirectly cause additional long-term adversity. Despite this, few population-wide studies have explored educational outcomes among schoolchildren with a previous cancer diagnosis and none have been conducted in the UK. Five educational outcomes were of interest for this study: special educational need (SEN), absenteeism, exclusion, academic attainment, and unemployment among school leavers. To the best of our knowledge, this is the first study worldwide to investigate exclusion, and the first to simultaneously investigate as wide a range of educational outcomes.

## Methods

### Databases

Individual-level data were obtained from four national educational databases held by the Scottish Government and five national health databases held by Public Health Scotland. These datasets were previously linked using individual-level identifiers [7, 8]. The Scottish Candidate Number (SCN) is an identifier allocated to all pupils attending a Scottish local-authority school, identifying pupils within educational datasets [9]. The Community Health Index (CHI) is an identifier given to every person registered with a Scottish NHS general practice, identifying individuals within health datasets [10]. Probabilistic matching linked each pupil’s SCN and CHI, generating a combined dataset covering educational outcomes and health data for each pupil.

The Scottish School Pupil Census is an annual national register of all children currently attending local-authority run primary, secondary, and special schools in Scotland and includes information on pupils’ demographics and any special educational needs [11]. Absences and exclusions recorded by schools are appended to the pupil census at the end of each year [12]. Individual-level data on examinations are collected by the Scottish Qualifications Authority [13]. The School Leaver Status Survey collect information on pupils’ employment and education status 6 months after leaving school [14].

The Scottish Morbidity Registry (SMR) 02 Maternity Inpatient Database contains national data on obstetric, child, and maternity outcomes [15]. The SMR06 Scottish Cancer Registry is a national dataset of all cancer diagnoses [16]. SMR01 and SMR11 record admissions to general acute wards and neonatal units respectively. The Scottish Birth record is a Scotland-wide neonatal data collection that replaced SMR11 in 2002.Within all SMR databases, diseases are recorded using the International Classification of Diseases versions 9 and 10 (ICD-9/ICD-10). The Prescribing Information System (PIS) contains national data on prescriptions dispensed by community pharmacists or via primary care [17].

### Inclusion criteria and definitions

The study population included all singleton children born in Scotland who were enrolled at local-authority primary, secondary, or special schools in Scotland at any time between 2009–2013 inclusive. Five years of data were available for each pupil unless they joined or left school during these years. We excluded: births involving multiple offspring as the children could not be reliably matched to their birth records; pupils aged <4 or ≥19 years at the time of the School Pupil Census; and pupils whose date of cancer diagnosis was recorded as either on or before their date of birth (deemed an error).

Final school attainment and unemployment were limited to pupils leaving school within the study period. SEN, absenteeism, and exclusion were analysed for each pupil as annual outcomes. Due to a national change in data collection, absenteeism and exclusion data were only available for 2009, 2010, and 2012. SEN describes support given to pupils above what is normally provided and is assessed by teachers and other school staff [18] and was attributed to one or more of the following: autism; learning disability; learning difficulty; sensory impairment; physical motor disability; communication problems; social, emotional, or behavioural difficulty; mental health condition, or physical health condition. Absenteeism and exclusion (for inappropriate behaviour) were assessed using the number of days absent per year (authorised and unauthorised combined) and number of exclusions per year, respectively. SQA examinations are typically taken during the fourth, fifth, and sixth years of secondary school (ages 15–18 years) [19]. Level of attainment over the last three years of secondary school was derived from the number of examination grades attained at each level of the Scottish Credit and Qualifications Framework (SCQF) [20]. Attainment was then dichotomised as low attainment (≥1 at SCQF level 2, ≥5 at SCQF level 3, ≥2 but ≤ 7 at SCQF level 4, or >0 but ≤4 at SCQF level 5) or high attainment (>7 at SCQF level 4, ≥5 at SCQF level 5, ≥3 at SCQF level 6, or ≥1 at SCQF level 7). Unemployment was defined as pupils not in education, employment (including voluntary work), or training (NEET) six months after leaving school.

Pupils were ascertained as having a previous cancer diagnosis if they had any record of an SMR06 cancer registration with ICD10 codes C00-D49 (neoplasms) prior to the school year being analysed. Whilst ICD10 replaced ICD9 in 1997, diagnoses prior to this date using ICD9 were mapped to ICD10 on the cancer registry. Children were further ascertained as having a previous haematological cancer or a previous cancer relating to the central nervous system (CNS) as these are the most commonly occurring cancers in childhood [1] and contained sufficiently large sample sizes. Furthermore, children with cancers of the central nervous system are thought to be more susceptible to poorer educational outcomes [21–26]. Haematological cancers were defined as an ICD-10 code C81-C96 (malignant neoplasms, stated or presumed to be primary, of lymphoid, haematopoietic and related tissue) [27]. Central Nervous System cancers were defined as ICD-10 codes C69-C72 (malignant neoplasms of eye, brain, and other parts of central nervous system).

Four models were used: unadjusted; adjusted for child sociodemographic covariates; adjusted for child sociodemographic and maternity covariates; and adjusted for child sociodemographic and maternity factors, and chronic conditions (including any previous record of a congenital anomaly). Sociodemographic confounders were pupil age, sex, ethnicity, and deprivation using Scottish Index of Multiple Deprivation (SIMD) 2012 [28] population quintiles derived from current postcode of residence. Maternity factors, some of which have previously been associated with poor educational outcomes [29, 30], were maternal age, estimated gestation period, sex-gestation-specific birthweight centiles, smoking status during pregnancy, parity, mode of delivery, and 5-minute Apgar score. Chronic conditions previously associated with poor educational outcomes [8, 31–35] were ascertained from dispensed prescriptions for asthma (inhaled corticosteroid), depression (tricyclic antidepressant, selective serotonin reuptake inhibitor, mirtazapine, or venlafaxine), epilepsy (any drug from BNF section 4.8), [36] diabetes (insulin), attention deficit hyperactivity disorder (ADHD) (methylphenidate hydrochloride, dexamphetamine sulphate, atomoxetine or lisdexamfetamine dimesylate), and skin disorders (emollient, topical corticosteroid, or preparations for eczema or psoriasis). Records of any previous congenital anomalies were identified and defined as any SMR01, SMR02, SBR or SMR11 episode with a relevant ICD code, defined as ICD-9 740–759 or ICD-10 Q20-Q28.

### Statistical analyses

Analyses were conducted using *Stata/MP 16*.*0 for Windows*. Data were accessed and analysed securely via the National Safe Haven managed by Public Health Scotland.

The characteristics of children previously diagnosed with cancer were compared with their peers using Pearson’s χ^2^ test for association and trend for non-ordered and ordered categorical data, respectively, and an independent-samples t-test for continuous data. Absenteeism, number of exclusions, and SEN were assessed as annual outcomes across available years. As multiple records from the same pupil were included, generalised estimating equations (GEE) were used to adjust for correlations between records from the same pupil [37]. The most appropriate correlation structure was selected using the ‘Quasi-Likelihood under the Independence Model Criterion’ statistic [38].

GEE logistic regression models with a binomial distribution and logit link function were used to assess relationships between previous cancer and SEN to calculate odds ratios (OR). The odds of having any form of SEN, and then each specific type of SEN, were investigated using separate models. Number of days absent, and number of exclusions were analysed using GEE analyses with a negative binomial distribution and log-link function to calculate incidence rate ratios (IRR). Both outcomes were adjusted for the offset variable of number of possible days attended annually to account for different exposure times. Unemployment and academic attainment were investigated using binary logistic regression models to produce odds ratios.

Firstly, pupils with any previous cancer diagnosis were compared to pupils who had never been diagnosed with cancer to investigate the associations with all the educational outcomes. Interactions with pupil sex were examined for each outcome and subgroup analyses by pupil sex were performed where significant interactions (p<0.05) were found.

Secondly, the same models were re-run comparing pupils with a previous haematological cancer, and pupils with a previous CNS cancer, to pupils who had never been diagnosed with cancer. Children with non-haematological cancers and children with non-CNS cancers were omitted. Finally, in the sub-group of children with previous cancer diagnoses, models were run to test the associations between age at diagnosis and educational outcomes, time since diagnosis and educational outcomes, and type of treatment and educational outcomes. Age at diagnosis was categorised as primary school (5–11 years) and secondary school (≥18 years) referent to diagnosis before school (<5 years). Time since diagnosis was categorised as 1–5 years ago and >5 years ago referent to diagnosis within the last year. Children could have received more than one type of treatment therefore each treatment was included as a separate binary variable in the multivariate model: chemotherapy (yes v no), radiotherapy (yes v no), surgery (yes v no), other form (yes v no)

### Approvals

Approval for the study was obtained from the Public Benefit and Privacy Panel of Public Health Scotland (reference 1920–0144). A data processing agreement was drafted between Glasgow University and Public Health Scotland and a data sharing agreement between Glasgow University and ScotXed.

### Ethics

The NHS West of Scotland Research Ethics Service confirmed that formal NHS ethics approval was not required since the study involved anonymised extracts of routinely collected data with an acceptably negligible risk of identification.

## Results

Of 766,217 pupils born in Scotland who attended a Scottish local-authority school between 2009 and 2013, 1,313 (0.2%) had a previous cancer diagnosis (Table 1). Of the children diagnosed with cancer, 535 (40.7%) of children had been diagnosed with a haematological cancer and 247 (18.8%) had been diagnosed with a CNS cancer (S1 Table). Compared with their peers, children previously diagnosed with cancer were more likely to be male and more likely to be prescribed medication for diabetes, epilepsy, ADHD, depression, and skin disorders. (Table 1).

**Table 1:** Characteristics among children previously diagnosed with any cancer compared to peers.

Characteristic	No Previous Cancer		Previous Cancer*		Total		p-value^†^
	n = 764,904		n = 1,313		n = 766,217		
	N	%	N	%	N	%	
Average age (years) across all school years attended							
Mean (SD)	10.9 (3.97)		11.89 (3.84)		10.9 (3.97)		<0.001
Sex							
Male	389,562	50.9	714	54.4	390,276	50.9	0.012
Female	375,342	49.1	599	45.6	375,941	49.1	
SIMD Deprivation Quintile							
1 (most deprived)	173,517	22.7	271	20.6	173788	22.7	0.416
2	153,283	20.1	280	21.3	153,563	20.1	
3	147,653	19.3	264	20.1	147,917	19.3	
4	149,271	19.5	252	19.2	149,523	19.5	
5 (least deprived)	140,586	18.4	246	18.7	140,832	18.4	
Missing	594		0		594		
Ethnicity							
White	724,188	96.2	1242	96.4	725,430	96.2	0.807
Asian	17,682	2.3	28	2.2	17,710	2.3	
Black	1,929	0.3	5	0.4	1,934	0.3	
Mixed	6,678	0.9	9	0.7	6,687	0.9	
Other	1,985	0.3	4	0.3	1,989	0.3	
Missing	12,442		25		12,467		
Gestation at delivery (weeks)							
<34	11,521	1.5	20	1.5	11,541	1.5	0.08
34	5,751	0.8	12	0.9	5,763	0.8	
35	9,288	1.2	18	1.4	9,306	1.2	
36	17,162	2.2	37	2.8	17,199	2.2	
37	37,550	4.9	69	5.3	37,619	4.9	
38	95,803	12.5	186	14.2	95,989	12.5	
39	158,473	20.7	260	19.8	158,733	20.7	
40	230,049	30.1	375	28.6	230,424	30.1	
41	170,899	22.4	288	21.9	171,187	22.4	
≥42	27,847	3.6	47	3.6	27,894	3.6	
Missing	561		1		562		
Maternal age (years)							
≤24	209,534	27.4	338	25.7	209872	27.4	0.645
24–29	224,127	29.3	401	30.5	224,528	29.3	
30–34	216,546	28.3	387	29.5	216,933	28.3	
≥35	114,685	15	187	14.2	114,872	15	
Missing	12		0		12		
Smoked During Pregnancy							
No	490,253	72.3	844	72.9	491,097	72.3	0.772
Yes	187,470	27.7	313	27.1	187,783	27.7	
Missing	87,181		156		87,337		
Parity							
0	345,047	45.3	606	46.3	345,653	45.3	0.523
1	263,684	34.6	445	34	264,129	34.6	
>1	152,311	20	257	19.6	152,568	20	
Missing	3,862		5		3,867		
Mode of Delivery							
Spontaneous Vaginal Delivery	499,067	65.2	831	63.3	499,898	65.2	0.477
Cephalic	16,281	2.1	26	2	16,307	2.1	
Assisted Vaginal Delivery	91,483	12	169	12.9	91,652	12	
Breech	2,226	0.3	7	0.5	2,233	0.3	
Elective Caesarean	58,216	7.6	96	7.3	58,312	7.6	
Emergency Caesarean	97,466	12.7	184	14	97,650	12.7	
Other	163	0	0	0	163	0	
Missing	2		0		2		
Sex-Gestation-Specific Birthweight Centiles							
1 to 3	31,425	4.1	59	4.5	31,484	4.1	0.224
4 to 10	68,545	9	97	7.4	68,642	9	
11 to 20	91,195	11.9	150	11.4	91,345	11.9	
21 to 80	449,334	58.8	775	59.1	450,109	58.8	
81 to 90	65,233	8.5	128	9.8	65,361	8.5	
91 to 97	41,142	5.4	78	5.9	41,220	5.4	
98 to 100	17,052	2.2	25	1.9	17,077	2.2	
Missing	978		1		979		
5-Minute Apgar score							
1 to 3	3,699	0.49	10	0.8	3,709	0.5	0.057
4 to 6	7,285	1	17	1.3	7,302	1	
7 to 10	746,108	98.5	1278	97.9	747,386	98.5	
Missing	7,812		8		7,820	1	
Diabetes							
No	761,587	99.6	1300	99	762,887	99.6	0.002
Yes	3,317	0.4	13	1	3,330	0.4	
Asthma							
No	719,078	94	1,240	94.4	720,318	94	0.511
Yes	45,826	6	73	5.6	45,899	6	
Epilepsy							
No	759,701	99.3	1,205	91.8	760,906	99.3	<0.001
Yes	5,203	0.7	108	8.2	5,311	0.7	
ADHD							
No	757,517	99	1,288	98.1	758,805	99	0.001
Yes	7,387	1	25	1.9	7,412	1	
Depression							
No	759,598	99.3	1,277	97.3	760,875	99.3	<0.001
Yes	5,306	0.7	36	2.7	5,342	0.7	
Skin Disorder							
No	635,104	83	1,031	78.5	636,135	83	<0.001
Yes	129,800	17	282	21.5	130,082	17	
Congenital Anomaly							
No	717,957	93.9	1,140	86.8	719,097	93.9	<0.001
Yes	46,947	6.1	173	13.2	47,120	6.1	

* Includes all pupils diagnosed with cancer before the end of the study period

^†^ p-values were calculated as follows: **Pearson’s χ**^**2**^ **Test of Association:** Sex, Ethnicity, Smoking Status During Pregnancy, Mode of Delivery, Diabetes, Asthma, Epilepsy, ADHD, Depression, Skin Disorder, Autism, Learning Disability, Learning Difficulty, Sensory Impairment, Physical and Motor Problems, Communication Problems, Social, Emotional, or Behavioural Difficulty, Physical Health Condition, Mental Health Condition, Any Special Educational Need, Congenital Anomaly. **Pearson’s χ**^**2**^ **Test of Trend:** SIMD, Parity, Estimated Gestation Period, Maternal Age Category at Birth, Sex-Gestation-Specific Birthweight Centile, Apgar score. **Independent-samples t-test:** Average Age Across Years

### Special educational need

Children with any previous cancer diagnosis had greater risk of having a record of SEN compared to their peers after adjusting for sociodemographic, maternity, and comorbid condition confounders (OR 3.26, 95% CI 2.86–3.71) (Table 2). This association was observed among children with previous haematological cancer (OR 2.62, 95% CI 2.12–3.24) but stronger among children previously diagnosed with a CNS cancer (OR 6.44, 95% CI 4.91–8.46) compared to their peers with no cancer diagnosis after adjusting for sociodemographic, maternity, and comorbidity confounders (Table 3). There was a significant interaction with pupil sex (p<0.001) whereby the association between any previous cancer diagnosis and SEN was present in both sexes but stronger among girls (OR 3.91, 95% CI 3.23–4.72) than boys (OR 2.85, 95% CI 2.39–3.39) after full adjustment for confounders.

**Table 2:** Associations between any previous cancer diagnosis (versus no cancer diagnosis) and educational outcomes.

	Univariate			Multivariate 1^†^			Multivariate 2^‡^			Multivariate 3^§^		
	OR	95% CI	p value	OR	95% CI	p value	OR	95% CI	p value	OR	95% CI	p value
SEN	3.71	3.31–4.17	<0.001	3.76	3.33–4.24	<0.001	3.86	3.42–4.37	<0.001	3.26	2.86–3.71	<0.001
Low Academic Attainment	1.26	0.99–1.61	0.065	2.30	1.63–3.24	<0.001	2.45	1.74–3.45	<0.001	2.15	1.52–3.03	<0.001
Unemployment	1.17	0.89–1.55	0.268	1.24	0.93–1.65	0.148	1.28	0.96–1.71	0.092	1.17	0.88–1.57	0.287
	**IRR**	** **	** **	**IRR**	** **	** **	**IRR**	** **	** **	**IRR**		** **
Absenteeism	1.83	1.72–1.95	<0.001	1.86	1.74–1.99	<0.001	1.90	1.78–2.03	<0.001	1.82	1.70–1.94	<0.001
Exclusion	0.56	0.35–0.91	<0.05	0.50	0.31–0.80	<0.01	0.50	0.31–0.82	<0.01	0.51	0.31–0.83	<0.01

OR Odds Ratio; SEN: special educational need; CI Confidence Interval; IRR Incidence Rate Ratio

^†^ Adjusted for sociodemographic (age, gender, deprivation quintile, ethnicity) confounders

^‡^ Adjusted for sociodemographic (age, gender, deprivation quintile, ethnicity) and maternity (maternal age at birth, estimated gestation period, sex-gestation-specific birthweight centiles, smoker status during pregnancy, parity, mode of delivery, 5-minute Apgar score) confounders

^§^ Adjusted for sociodemographic (age, gender, deprivation quintile, ethnicity), maternity (maternal age at birth, estimated gestation period, sex-gestation-specific birthweight centiles, smoker status during pregnancy, parity, mode of delivery, 5-minute Apgar score), and comorbid condition (diabetes, asthma, epilepsy, ADHD, depression, skin disorder, previous congenital anomaly) confounders

**Table 3:** Association between any previous haematological or CNS cancer diagnosis (versus no cancer diagnosis) and educational outcomes.

	Univariate Model			Multivariate Model 1^†^			Multivariate Model 2^‡^			Multivariate Model 3^§^		
	**Effect Size**	**95% CI**	**p value**	**Effect Size**	**95% CI**	**p value**	**Effect Size**	**95% CI**	**p value**	**Effect Size**	**95% CI**	**p value**
	**OR**	** **	** **	**OR**	** **	** **	**OR**	** **	** **	**OR**		** **
SEN												
Haematological	2.82	2.34–3.40	<0.001	2.75	2.26–3.35	<0.001	2.84	2.32–3.49	<0.001	2.62	2.12–3.24	<0.001
CNS	7.40	5.86–9.34	<0.001	8.15	6.40–10.38	<0.001	8.43	6.62–10.74	<0.001	6.44	4.91–8.46	<0.001
Low Academic Attainment												
Haematological	1.08	0.75–1.56	0.676	2.04	1.23–3.38	<0.01	2.38	1.44–3.95	<0.001	2.17	1.31–3.61	<0.01
CNS	1.84	1.05–3.22	<0.05	4.07	1.88–8.82	<0.001	4.39	2.03–9.47	<0.001	3.33	1.52–7.30	<0.01
Unemployment												
Haematological	1.34	0.91–1.98	0.143	1.39	0.93–2.08	0.112	1.49	1.00–2.24	0.052	1.39	0.92–2.09	0.114
CNS	0.67	0.27–1.67	0.391	0.68	0.27–1.73	0.421	0.71	0.28–1.82	0.481	0.58	0.23–1.49	0.258
	**IRR**	** **	** **	**IRR**	** **	** **	**IRR**	** **	** **	**IRR**		** **
Absenteeism												
Haematological	1.95	1.78–2.14	<0.001	1.97	1.79–2.16	<0.001	2.08	1.88–2.29	<0.001	2.04	1.85–2.25	<0.001
CNS	1.77	1.52–2.07	<0.001	1.89	1.61–2.21	<0.001	1.91	1.64–2.22	<0.001	1.75	1.51–2.04	<0.001
Exclusion												
Haematological	0.57	0.28–1.15	0.115	0.46	0.23–0.92	<0.05	0.52	0.26–1.04	0.063	0.50	0.25–1.01	0.053
CNS	0.23	0.09–0.62	<0.01	0.26	0.10–0.68	<0.01	0.2	0.06–0.61	<0.01	0.20	0.06–0.61	<0.01

OR Odds Ratio; SEN: special educational need; CI Confidence Interval; IRR Incidence Rate Ratio

^†^ Adjusted for sociodemographic (age, gender, deprivation quintile, ethnicity) confounders

^‡^ Adjusted for sociodemographic (age, gender, deprivation quintile, ethnicity) and maternity (maternal age at birth, estimated gestation period, sex-gestation-specific birthweight centiles, smoker status during pregnancy, parity, mode of delivery, 5-minute Apgar score) confounders

^§^ Adjusted for sociodemographic (age, gender, deprivation quintile, ethnicity), maternity (maternal age at birth, estimated gestation period, sex-gestation-specific birthweight centiles, smoker status during pregnancy, parity, mode of delivery, 5-minute Apgar score), and comorbid condition (diabetes, asthma, epilepsy, ADHD, depression, skin disorder, previous congenital anomaly) confounders

Pupils with a previous cancer diagnosis were more likely to have SEN specifically attributed to learning disability, learning difficulty, sensory impairment, physical motor difficulty, communication problems, and physical health condition compared to pupils without cancer, after adjustment for sociodemographic and maternity variables and comorbid conditions (S2 Table). Among children with a previous cancer, risk of SEN was partially associated with age at diagnosis, increased with time since diagnosis, and increased when treatment included radiotherapy (Table 4).

**Table 4:** Association between age at diagnosis, time since diagnosis, and treatment modality and special educational need/absenteeism among children previously diagnosed with any cancer.

	Univariate			Multivariate 1^†^			Multivariate 2^‡^			Multivariate 3^§^		
**SEN**												
	**OR**	**95% CI**	**p value**	**OR**	**95% CI**	**p value**	**OR**	**95% CI**	**p value**	**OR**	**95% CI**	**p value**
Age At Diagnosis (Years)												
< 5 (ref.)	1.00			1.00			1.00			1.00		
5–11	1.30	1.02–1.66	<0.05	1.30	1.01–1.68	<0.05	1.26	0.97–1.62	0.084	1.24	0.95–1.61	0.114
≥ 12	0.62	0.43–0.88	<0.01	0.65	0.44–0.97	<0.05	0.66	0.44–0.98	<0.05	0.58	0.38–0.88	<0.05
Time Since Diagnosis (Years)												
< 1 (ref.)	1.00			1.00			1.00			1.00		
1–5	1.50	1.18–1.91	<0.001	1.43	1.12–1.82	<0.01	1.45	1.13–1.87	<0.01	1.50	1.15–1.94	<0.01
≥ 5	1.59	1.21–2.11	<0.01	1.61	1.21–2.14	<0.001	1.67	1.24–2.24	<0.001	1.78	1.31–2.41	<0.001
Treatment modality												
Chemotherapy	0.82	0.61–1.10	0.188	0.79	0.59–1.06	0.115	0.85	0.63–1.15	0.284	0.90	0.67–1.23	0.514
Surgery	0.96	0.73–1.27	0.795	0.98	0.74–1.30	0.883	1.02	0.77–1.36	0.897	1.02	0.77–1.36	0.871
Radiotherapy	1.98	1.44–2.73	<0.001	2.06	1.49–2.86	<0.001	2.07	1.49–2.89	<0.001	2.03	1.45–2.83	<0.001
Other therapy	0.79	0.54–1.16	0.238	0.77	0.52–1.13	0.183	0.74	0.49–1.11	0.148	0.73	0.50–1.09	0.124
**Absenteeism**												
	**IRR**	**95% CI**	** **	**IRR**	**95% CI**	** **	**IRR**	**95% CI**	** **	**IRR**	**95% CI**	** **
Age at Diagnosis (Years)												
< 5 (ref.)	1.00			1.00			1.00			1.00		
5–11	1.61	1.40–1.84	<0.001	1.63	1.43–1.87	<0.001	1.60	1.40–1.83	<0.001	1.56	1.36–1.78	<0.001
≥ 12	2.28	1.91–2.71	<0.001	2.44	2.01–2.96	<0.001	2.44	2.01–2.98	<0.001	2.35	1.93–2.87	<0.001
Time Since Diagnosis (Years)												
< 1 (ref.)	1.00			1.00			1.00			1.00		
1–5	0.48	0.41–0.55	<0.001	0.50	0.43–0.58	<0.001	0.5	0.43–0.59	<0.001	0.49	0.42–0.57	<0.001
≥ 5	0.34	0.29–0.40	<0.001	0.32	0.27–0.37	<0.001	0.33	0.28–0.38	<0.001	0.33	0.28–0.39	<0.001
Treatment modality												
Chemotherapy	1.13	0.96–1.32	0.146	1.13	0.97–1.33	0.117	1.16	0.99–1.36	0.073	1.14	0.97–1.34	0.102
Surgery	0.93	0.80–1.09	0.400	0.91	0.78–1.05	0.204	0.90	0.77–1.04	0.153	0.89	0.76–1.03	0.125
Radiotherapy	1.14	0.94–1.39	0.185	1.15	0.96–1.39	0.137	1.08	0.90–1.29	0.396	1.03	0.83–1.23	0.754
Other therapy	1.67	1.37–2.03	<0.001	1.59	1.31–1.92	<0.001	1.58	1.30–1.92	<0.001	1.57	1.29–1.92	<0.001

OR Odds Ratio; SEN: special educational need; CI Confidence Interval; IRR Incidence Rate Ratio

^†^ Adjusted for sociodemographic (age, gender, deprivation quintile, ethnicity) confounders

^‡^ Adjusted for sociodemographic (age, gender, deprivation quintile, ethnicity) and maternity (maternal age at birth, estimated gestation period, sex-gestation-specific birthweight centiles, smoker status during pregnancy, parity, mode of delivery, 5-minute Apgar score) confounders

^§^ Adjusted for sociodemographic (age, gender, deprivation quintile, ethnicity), maternity (maternal age at birth, estimated gestation period, sex-gestation-specific birthweight centiles, smoker status during pregnancy, parity, mode of delivery, 5-minute Apgar score), and comorbid condition (diabetes, asthma, epilepsy, ADHD, depression, skin disorder, previous congenital anomaly) confounders

### Absenteeism

Among the 766,217 pupils with valid attendance data, pupils diagnosed with any cancer had more days absent than their peers after adjusting for sociodemographic, maternity, and comorbidity confounders (OR 1.82, 95% CI 1.70–1.94) (Table 2). Absenteeism was greater among pupils with both haematological (IRR 2.04, 95% CI 1.85–2.25) and CNS (IRR 1.75, 95% CI 1.51–2.04) cancers compared to pupils without cancer, after adjusting for sociodemographic, maternity, and comorbidity factors (Table 3). There was no interaction with pupil sex (p = 0.177). Among children with a previous cancer, absenteeism decreased with increased time since diagnosis, increased with age at diagnosis and increased when cancer treatment comprised other forms (such as immunotherapy or stem cell transplants) in addition to, or in place of, chemotherapy, radiotherapy, or surgery. (Table 4).

### Exclusions

Among the 766,217 pupils with valid attendance data, previous cancer diagnosis was associated with lower risk of exclusion after adjusting for sociodemographic, maternity, and comorbidity factors (OR 0.51, 95% CI 0.31–0.83) (Table 2). This overall association was driven by pupils with CNS cancer (IRR 0.20, 95% CI 0.06–0.61) (Table 3). No association was observed among pupils diagnosed with haematological cancers. There was no interaction with pupil sex (p = 0.200). Associations with age at diagnosis, time since diagnosis, and type of treatment among children with previous cancer could not be assessed due to small sample size.

### Academic attainment

Among 139,200 children with attainment data, those with a previous cancer diagnosis were more than twice as likely to have low exam attainment after adjusting for sociodemographic, maternity, and comorbidity factors (OR 2.15, 95% CI 1.52–3.03) (Table 2). Associations with low attainment were observed for both haematological (OR 2.17, 95% CI 1.31–3.61) and CNS (OR 3.33, 95% CI 1.52–7.30) cancers compared to peers with no previous cancer, after full adjustment for confounders (Table 3). There was no interaction with pupil sex (p = 1.000). Associations with age at diagnosis, time since diagnosis, and type of treatment among children with previous cancer could not be assessed due to small sample size.

### Unemployment

Analyses of the 217,915 school leavers found no significant associations with unemployment six months after leaving school for cancer overall (Table 2) or haematological or CNS cancers specifically (Table 3).

## Discussion

This was the first non-selective, population-wide cohort study to investigate five educational outcomes among children previously diagnosed with cancer compared to their peers. Pupils with a previous cancer diagnosis were found to have increased risk of SEN, absenteeism, and lower academic attainment; decreased risk of exclusion; but no difference in employment upon leaving school compared to peers without cancer.

Increased SEN is consistent with results from previous studies [21–23, 39–42]. A meta-analysis [23] including nine studies found risk of SEN was over two-fold higher compared to matched controls or siblings (OR 2.47, 95% CI 1.91–3.20). There was significant heterogeneity between the studies (I^2^ = 52%, p = 0.02), thought to be due to differences in how SEN was assessed and defined between countries. In our study, risk of SEN was greater for children with CNS cancers compared to haematological cancers. This suggests that CNS cancers or their treatment may impact directly on cognition, which is consistent with previous literature [21, 22]. Lower risk of SEN among children diagnosed in secondary school compared to pre-school suggests a greater impact of cancer on children during early development. Risk of SEN among cancer survivors increased with time since diagnosis however this may simply be attributable to diagnosis at a younger age being correlated with increased time since diagnosis. SEN was also more likely among children who had been treated with radiotherapy which may be due to cranial irradiation being more likely to cause cognitive impairment and/or learning difficulties. The previous meta-analysis reported no association between type of cancer or treatment, age at diagnosis, and time since diagnosis. However, two Canadian studies, included in the meta-analysis, [21, 22] reported greater risk of SEN among children with CNS cancer compared to all-cause cancer.

Our finding of increased absenteeism is also consistent with previous studies. A Canadian cross-sectional study [3] reported greater absenteeism among 131 cancer survivors diagnosed at least 4 years previously compared to 167,752 peers without cancer. Only 13% of absent days were due to healthcare appointments, suggesting most absenteeism is for other reasons. Absenteeism was greater among children with brain tumours compared to those with haematological or solid organ cancers, which was attributed to more hospital appointments. Absenteeism was also greater among children who had been treated with alternative therapies such as immunotherapy or stem cell transplantation. This may be due to the duration of treatment, side-effects from the treatment, or the need to isolate.

Previous studies reported inconsistent findings as to how a previous cancer diagnosis affected academic attainment [43]. Whilst previous studies agreed that educational outcomes were worse among pupils with CNS cancer [23–25, 44], findings for haematological or all-cause cancers were less consistent. These variances have been recognised in a recent systematic review attributing inconsistent findings to the paucity of high-quality population studies [43].

This study determined that lower attainment was seen in pupils with all-cause, haematological, and CNS cancers, but only after adjusting for sociodemographic and maternity factors. Further population-level research would be beneficial to corroborate these findings and investigate other influencing factors, such as the school year cancer was diagnosed.

Having a previous cancer diagnosis did not place pupils at an increased risk of unemployment 6 months after leaving school. This was consistent with another study assessing employment in this age group [45], suggesting that the adverse effects of cancer on educational outcomes may decrease over time.

This was the first study investigating school exclusion amongst children with cancer. Possible reasons for lower exclusion among pupils with a previous CNS cancer compared to peers include less misconduct as a sequela of CNS treatment, differences in teacher behaviour, or simply residual confounding. It is unlikely to be solely due to greater absenteeism, as the effect was not observed among children with a haematological cancer. Further research into the reasons for lower school exclusion would be helpful.

### Strengths and limitations

Ours was a non-selective, population-wide study, in which children were identified through schools rather than healthcare services. This ensured sufficient statistical power for several subgroup analyses and ensured inclusion was not restricted to the most severe cancer cases. Whilst only pupils from local authority schools were included, this encompassed 95% of the school population in Scotland. Data linkage methods were robust and are less susceptible to recall bias. This study adjusted for four sociodemographic, seven maternity, and six comorbidity confounders. Whilst some residual confounding may remain, the ability to adjust for such a broad range of covariates greatly enhanced the validity of results. Some genetic conditions can result in learning difficulties whilst also predisposing to cancer. Whilst we were not able to identify specific genetic conditions, we were able to identify children with any previous congenital anomaly, including conditions such as Downs syndrome, and could adjust for these in our fully adjusted models. Data were limited to pupils’ first cancer diagnosis and data on cancer severity and treatment duration were not available and should be the focus of future work. Consequently, whilst time since first diagnosis was known, time from treatment completion was not, and the impact of subsequent cancer relapses, including secondary cancers of a different type, could not be considered. Since SEN was recorded at school, for incident cancers occurring before school age it was impossible to determine whether the underlying reasons for subsequent SEN were already present before the cancer. Additionally, in some cases where the SEN was diagnosed after the cancer, the underlying difficulties necessitating SEN may have been present for a period beforehand. Therefore, for the specific outcome of SEN, reverse causation may be a limitation.

As age of diagnosis and time since diagnosis were associated with absenteeism and SEN, it is reasonable to hypothesise that these factors may also influence exclusion rates, academic attainment, and unemployment however these analyses could not be conducted due to small sample sizes. Whilst unemployment six months after leaving school could be studied, data on longer-term employment status were not available. Longer term trajectories should be investigated in future studies.

Investigation of the association between treatment modality and exclusion, attainment and unemployment were also not possible within the cohort of children experiencing cancer due to sample size issues. However, we were able to investigate the impact of treatment modality on SEN and absenteeism. Radiotherapy treatment was associated with increased SEN and alternative treatments such as immunotherapy or stem cell transplantation were associated with increased absenteeism. Future, more thorough, investigation of the impact of different treatment modalities would help to further understand the extent to which cancer or cancer treatment drive the impact on educational outcomes.

Considering the limitations highlighted, future work should focus on understanding how educational outcomes are affected by factors beyond the scope of this study, including type and duration of treatment, cancer severity, and secondary cancers. This study highlights wider impacts of a childhood cancer diagnosis on educational outcomes. From a policy perspective, considering the poorer outcomes observed, these children need to be supported, as poorer educational outcomes can impact later health.

## Supporting information

S1 Table Characteristics among subgroup of children previously diagnosed with any cancer. (DOCX)

S2 Table Associations between any previous cancer diagnosis (versus no cancer diagnosis) and type of special educational need. (DOCX)
