# Supplementary material for: Educational outcomes in childhood cancer survivors: A Scotland-wide record-linkage study of 766,217 schoolchildren
Source: PLoS One. 2023 Jul 26;18(7):e0286840. doi: 10.1371/journal.pone.0286840 (PMC10370705; doi:10.1371/journal.pone.0286840)
Supplement: S1 Table — (DOCX) [file pone.0286840.s001.docx]

## Supplementary Table 1: Characteristics among subgroup of children previously diagnosed with any cancer

| Characteristic | No.  $\boldsymbol{n}\boldsymbol{=}\boldsymbol{1}\boldsymbol{,}\boldsymbol{313}$ | % |
| --- | --- | --- |
| Cancer Site |  |  |
| Benign Neoplasms | 33 | 2.5 |
| In Situ Neoplasms | 4 | 0.3 |
| Malignant Neoplasms of Bone and Articular Cartilage | 48 | 3.7 |
| Malignant Neoplasms of Breast and Female Genital organs | 18 | 1.4 |
| Malignant Neoplasms of Connective and Soft Tissue | 61 | 4.6 |
| Malignant Neoplasms of Digestive Organs | 18 | 1.4 |
| Malignant Neoplasms of Endocrine Glands and Related Structures | 59 | 4.5 |
| Malignant Neoplasms of Eye, Brain, and Central Nervous System | 247 | 18.8 |
| Malignant Neoplasms of Lip, Oral Cavity and Pharynx | 10 | 0.8 |
| Malignant Neoplasms of Male Genital Organs | 13 | 1.0 |
| Malignant Neoplasms of Respiratory System and Intrathoracic organs | 6 | 0.5 |
| Malignant Neoplasms of Skin | 23 | 1.8 |
| Malignant Neoplasms of Urinary organs | 76 | 5.8 |
| Malignant Neoplasms, Secondary and Ill Defined | 15 | 1.1 |
| Malignant Neoplasms, Stated or Presumed to be Primary, of Lymphoid, Haematopoietic and Related Tissue | 535 | 40.7 |
| Neoplasms of Uncertain or Unknown Behaviour | 147 | 11.2 |
|  | | |
| Age of Diagnosis (Whole Years) | | |
| 0 | 147 | 11.2 |
| 1 | 109 | 8.3 |
| 2 | 127 | 9.7 |
| 3 | 155 | 11.8 |
| 4 | 109 | 8.3 |
| 5 | 96 | 7.3 |
| 6 | 67 | 5.1 |
| 7 | 70 | 5.3 |
| 8 | 54 | 4.1 |
| 9 | 44 | 3.4 |
| 10 | 45 | 3.4 |
| 11 | 46 | 3.5 |
| 12 | 47 | 3.6 |
| 13 | 38 | 2.9 |
| 14 | 52 | 4.0 |
| 15 | 60 | 4.6 |
| 16 | 36 | 2.7 |
| 17 | 11 | 0.8 |
|  | | |
| Time Since Cancer Diagnosis at Last Census Date (Whole Years) * | | |
| 0 | 136 | 10.4 |
| 1 | 120 | 9.1 |
| 2 | 110 | 8.4 |
| 3 | 80 | 6.1 |
| 4 | 97 | 7.4 |
| 5 | 112 | 8.5 |
| 6 | 75 | 5.7 |
| 7 | 81 | 6.2 |
| 8 | 86 | 6.5 |
| 9 | 61 | 4.6 |
| 10 | 54 | 4.1 |
| 11 | 61 | 4.6 |
| 12 | 69 | 5.3 |
| 13 | 63 | 4.8 |
| 14 | 46 | 3.5 |
| 15 | 38 | 2.9 |
| ≥16 | 24 | 1.8 |
|  | | |
| Treated with Chemotherapy | | |
| No | 417 | 33.7 |
| Yes | 821 | 66.3 |
| Missing | 75 |  |
|  | | |
| Treated with Radiotherapy | | |
| No | 1,035 | 84.1 |
| Yes | 196 | 15.9 |
| Missing | 82 |  |
|  | | |
| Treated with Surgery | | |
| No | 654 | 52.9 |
| Yes | 583 | 47.1 |
| Missing | 76 |  |
|  | | |
| Treated with Hormone Therapy or Other Therapy | | |
| No | 1,076 | 87.6 |
| Yes | 152 | 12.4 |
| Missing | 85 |  |
|  | | |
| Any Treatment Recorded | | |
| No | 145 | 11.0 |
| Yes | 1,168 | 89.0 |

Table displays demographics for all pupils within the study cohort who were diagnosed with a cancer before the last year of analysis.

* Measured as the difference between the date of cancer diagnosis and the date of census collection for the pupil’s final year within the cohort
