# Supplementary material for: Educational outcomes in childhood cancer survivors: A Scotland-wide record-linkage study of 766,217 schoolchildren
Source: PLoS One. 2023 Jul 26;18(7):e0286840. doi: 10.1371/journal.pone.0286840 (PMC10370705; doi:10.1371/journal.pone.0286840)
Supplement: S2 Table — (DOCX) [file pone.0286840.s002.docx]

## Supplementary Table 2: Associations between any previous cancer diagnosis (versus no cancer diagnosis) and type of special educational need

| Type of SEN | Multivariate 2^†^ | | | Multivariate 3^‡^ | | |
| --- | --- | --- | --- | --- | --- | --- |
|  | **OR** | **95% CI** | **p value** | **OR** | **95% CI** | **p value** |
| Learning Disability | 4.80 | 3.83–6.03 | <0.001 | 3.18 | 2.40–4.22 | <0.001 |
| Learning Difficulty | 1.96 | 1.58–2.42 | <0.001 | 1.68 | 1.35–2.10 | <0.001 |
| Sensory Impairment | 15.23 | 11.99–19.33 | <0.001 | 10.31 | 7.64–13.92 | <0.001 |
| Physical Motor Difficulty | 11.23 | 8.84–14.26 | <0.001 | 6.76 | 4.82–9.48 | <0.001 |
| Communication Problems | 3.71 | 2.74–5.01 | <0.001 | 2.45 | 1.69–3.56 | <0.001 |
| Autism Spectrum Disorder | 1.80 | 1.13–2.87 | <0.05 | 1.31 | 0.79–2.18 | 0.300 |
| Social, Emotional, or Behavioural Disorder | 1.63 | 1.20–2.20 | <0.01 | 1.35 | 0.99–1.84 | 0.06 |
| Physical Health Condition | 26.86 | 22.98–31.39 | <0.001 | 21.77 | 17.59–26.94 | <0.001 |
| Mental Health Condition | 3.71 | 1.68–8.17 | <0.01 | 2.16 | 0.89–5.24 | 0.087 |

For each type of SEN, those with that SEN type are compared to children with no recorded SEN.

OR Odds Ratio; SEN: special educational need; CI Confidence Interval
^†^ Adjusted for sociodemographic (age, gender, deprivation quintile, ethnicity) and maternity (maternal age at birth, estimated gestation period, sex-gestation-specific birthweight centiles, smoker status during pregnancy, parity, mode of delivery, 5-minute Apgar score) confounders
^‡^ Adjusted for sociodemographic (age, gender, deprivation quintile, ethnicity), maternity (maternal age at birth, estimated gestation period, sex-gestation-specific birthweight centiles, smoker status during pregnancy, parity, mode of delivery, 5-minute Apgar score), and comorbid condition (diabetes, asthma, epilepsy, ADHD, depression, skin disorder, previous congenital anomaly) confounders
